# Prof Benchie and Dr Athena—A modern tragedy…

**DOI:** 10.1002/ebm2.8

**Published:** 2015-06-27

**Authors:** Malcolm Macleod

**Affiliations:** ^1^ Centre for Clinical Brain SciencesUniversity of EdinburghScotlandUK

## PROF BENCHIE AND DR ATHENA—A MODERN TRAGEDY …

…..in which Professor Benchie, an established super‐sub‐speciality clinician who spends most of his time performing laboratory research, profers advice to Athena, an enthusiastic early career physician who, although she spent summers in the lab as a medical student, is just finishing a research fellowship that included a course in clinical research and wonders whether she would prefer to devote her research time to patient‐based therapeutic trials.

## HUBRIS

“*So Prof Benchie, my hero”, asked Dr Athena, the seeker after truth, “There are hundreds of diseases in this world which need a cure, and with my three year fellowship I am just the person to do it. Whither should I direct my research endeavours?*”

“I still can't tempt you to my laboratory? The clean white bench, the precision scales, the control of every variable? That's where the future lies, not in messy research involving human subjects who don't take their tablets, and who default from follow up, and where you need thousands of patients to say anything of substance. Why, I can show a drug improves pain symptoms using only 6 animals”

“*I know, I know, I'm really jealous of the purity of the laboratory but I'd really like to try doing research which helps people right away. What about taking something that works in the lab, and seeing whether it works in patients? That thing they call ‘translational medicine.’ So much has been written about it, there must be well validated approaches to doing that kind of thing?*”

“Well, here in the lab we tend not to get involved in that sort of methodology. Our job is to show that the drug can work in our model systems”

“*That the drug **can** work?*”

“Under the right circumstances, of course, with a model of the correct severity, in experienced hands, when the moon is in the correct phase and other heavenly bodies are appropriately distributed”

“*So, before I start my clinical trial to see whether this drug **does** work, is there anything I should try to find out about the bench research that nominated it; anything else I should know?*”

“Jeez, you're asking me? You're the one talking about wanting to do clinical trials. I guess though that there are a few things you could look out for. Firstly, do the *in vivo* data come from a good lab?”

“*How do I know if it's a good lab? You mean that it does high quality research at low risk of bias? Are there parallel standards to those applied in clinical trials? Stuff like randomised animals, blinded treatments and observations, pre‐specified primary analyses, appropriate sample size calculations, and the reporting of animals excluded from analyses?*”

“Look, don't start with that mumbo‐jumbo. You just know their results are true because you know their work, and have reviewed their papers and so you just know. None of your fancy biases apply in the lab, because we can control everything. All the animals are identical, and we measure outcomes using machines which are not susceptible to bias. If we lose an animal during an experiment we just add another, and if you use the right statistical test on the right subgroup you need hardly any animals”

“*Ok, lets come back to that later, because I'm not sure I share your confidence in bias‐free bench research. What else?*”

“Well, you'll want to know that the findings have been reported in another lab”

“*Great. Is finding that confirmation straightforward? I mean, is there something like* clinicaltrials.gov, *where I can find all the lab experiments that have tested the drug, so I can see if they have consistently positive findings?*”

“I'm afraid not, but you could look in Pubmed. The trouble (Professor Benchie shifts uneasily) is that some experiments might never have been published. And, it's possible that these unpublished studies are the ones that concluded the drugs don't work—but since they never got published we don't know. You'll just have to trust the research that **did** get published”

“*With all due respect, that's crazy! Don't you guys look for publication bias and measure its impact? And don't you look to see whether there's an outcome reporting bias by comparing, published results with their respective study protocols?”.*

“Protocols? You really don't understand how bench research works, do you? A publication should be a thing of rare beauty, flawless in every respect. We might start off with a protocol, but we'll end up doing something different. How that happens isn't important. So we might look at 4 or 5 different measures of outcome, and of course we'll report the ones that show significant effects. And we'll use a range of statistical tests according to what seems to fit the data best”

“*Prof, now I'm really worried. I'm not sure, from what you've said, that I can rely on conclusions that come out of the lab. Far from being clean, and precise, you seem to be ignoring problems with validity which, because they also bedevil clinical studies, we've worked to prevent and overcome. Let me go and read up about this ….”*

With this Dr Athena goes off to the biomedical library and does some literature research, helpfully guided by their staff and an expanding number of relevant publications on this issue ….

Later that same month ….

## NEMESIS

*…. OK, Prof Benchie, sit down and hear the news:*‐

1. First, it turns out that animal studies are just as vulnerable to bias as clinical studies. Worse yet, lots of them fail to avoid them. For example, non‐randomised, non‐blinded lab studies were five times as likely to report positive findings than randomised, blinded studies. Moreover, the repeated inability of other bench researchers to replicate these positive results shows that lots of the former had to be false‐positives. Across models of neurological disease there is a persistent overstatement of drug efficacy in studies that do not take simple measures to reduce the risk of bias.
2. A minority of animal studies—in neurological disease and more broadly—report taking these simple measures to reduce the risk of bias. Finally, appearance in journals with high impact factors carries no guarantee that investigators have made even the most basic efforts to reduce the risk of bias.
3. There's a lot of publication bias about too, and I couldn't find any systematic efforts by the *in vivo* community to address this issue. The best estimate from attempts to measure the scale of the problem is that around 20% of bench studies remain unpublished, and that translates to an overstatement of efficacy by around one third.
4. The whole field of *in vivo* research doesn't seem to know what a power calculation is—and many studies are underpowered for the effects they purport to detect. In stroke research, for instance, studies are powered at about 30%, so two‐thirds of research effort—including yours, Professor Benchie—is likely to fail even if a pre‐stated null hypothesis is false.
5. Because study protocols and their statistical analysis plans are not routinely available, we simply can't tell if either (1) the outcomes and their measures reported are those that the investigator had decided a priori to be the most important (rather than the result of looking for the most statistically significant pony) one; or (2) that the statistical test reported is the one specified prior to the study, or simply the first one which gave a *p* value of less than 0.05).^1^
6. In summary, my literature search documented that, when bench scientists attempt to replicate the work of their fellow bench scientists—for instance in drug discovery work, or cancer, or motor neuron disease models—they have not been able to replicate about two‐thirds of the positive findings originally reported. Indeed, this is often the case when the drugs being tested are nominated by robust systematic review and meta‐analysis.
7. No wonder, then, that of 374 compounds alleged to provide neuroprotection after stroke in animal models, only one generated positive evidence of efficacy in human RCTs.

“Cripes, Athena. Your systematic demolition of our cherished bench research methods has shaken my confidence in my own work, and I need to go away and read and think and reformulate my research strategies and tactics. I guess you won't be coming near the lab then”

Box 1 **What Athena found out**

- In 2003, Bebarta et al. reported^3^ an analysis of abstracts submitted to the Society for Academic Emergency Medicine that described research using cell lines or animals. They found that 252 of 290 studies reported statistically significant findings. 94 reported randomisation and only 31 reported blinding. Non randomised (odds ratio 3.4) and non‐blinded (odds ratio 3.2) studies were much more likely to report significant findings; and non‐randomised, non‐blinded studies were even more likely to report significant findings than the 10% of studies which were both randomised and, blinded studies (odds ratio 5.2).
- Dan Hackam looked at the fate of 76 interventions reported in highly cited publications in seven leading journals which investigated a preventative or therapeutic intervention in an *in vivo* animal model.^4^ 37% of studies had been replicated in human randomised trials and 18% were contradicted by such studies; 45% remained untested in humans.
- Despina Contopoulos‐Ioannidis identified 101 articles published between 1979 and 1983 in high impact basic science journals in which it was claimed that the technology studied had novel preventative or therapeutic potential. By 2002 five drugs were licenced for clinical use, but only one had entered into common use for the licenced indication.
- Tori O'Collins studied the fate of drugs developed for the treatment of ischaemic stroke;^5^ of 374 drugs which had some reports of efficacy in animal models of focal cerebral iscahemia, only one—clot‐busting treatment with tPA—had successfully translated to human health.
- Just about every systematic review of animal data shows low levels of reporting of those study design features which might reduce the risk of bias—across stroke,^6^, ^7^, ^8^ multiple sclerosis,^9^ Parkinson's disease,^10^ glioma,^11^ myocardial ischaemia,^12^ spinal cord injury,^13^ etc. … While reporting of randomisation and blinding in less than half of studies, sample size calculations—how the size of the experiment was chosen—is reported in less than 1% of studies. Initially this appeared to be a problem with the *in vivo* stroke literature, because that's where this work started—but in a random sample of *in vivo* and *in vitro* research in Pubmed randomisation was reported by only 14%, and the blinded assessment of outcome by only 2%.^14^
- ter et al. reported that most Dutch laboratory animal researchers considered publication bias to be a substantial problem, and estimated that around 50% of studies remained unpublished.^15^
- Using data from *in vivo* stroke modelling Sena et al. used standard statistical approaches to suggest that around one in six studies remained unpublished, leading to an overstatement of treatment efficacy of around 30%.^16^
- Tsilidis et al. studied the distribution of *p* values reported in 4445 *in vivo* experiments testing drug efficacy in animal models of neurological disease.^17^ They found a gross excess of statistically significant findings (1719, compared with an expected 919 positive studies), suggesting that, even once publication bias had been taken in to account, the outcomes from many thousands of individual experiments had not been reported—that is that there was selective outcome reporting bias.

## CATHARSIS

“*You know Benchie, I think I just might. Bench scientists are clearly in need of some help, and your responsiveness to my review suggests a receptivity to changes in the way you generate the bench research input into translational medicine by borrowing and employing the bias‐reducing strategies and tactics of RCTs. Collaborating in sorting that out could be both worthy and lots of fun!*”

“But what if—it pains me to even consider the possibility—what if it's all wrong, like the MND research, or the stroke drugs? How will you know that there's any prospect of success?”

“*We could replicate the approach of stroke trialists in your lab—conducting large, adequately powered, proof of concept animal studies at low risk of bias, according to strict protocols with pre‐specified primary outcome measures and pre‐specified statistical analysis plans. We might even think about doing multicentre animal studies, with central randomisation and outcome assessment, and monitoring to drive up standards and to detect fraud”.*

“Fraud? Are you saying that you people fabricate data, too?”

“*Some people do.* 1 *I'd like to think this is a very small proportion of the research community, although it can be difficult to detect so may be more prevalent. Central statistical monitoring in a multicentre study is what led to detection of fraud in the Darsee case,* 2 *and detailed in‐house review of raw data is what detected fraud in the Eaton case, where a scientist ended up in prison for falsifying data (* http://www.bbc.co.uk/news/uk‐scotland‐edinburgh‐east‐fife‐22186220 *).*

“I hope that is uncommon—I've never directly observed it in my colleagues”

“*Indeed. The overwhelming majority of scientists—in the lab or the clinic—want to do research of the highest quality. It's just that over the years, these efforts have been subverted through worship of the false Gods ‘grants in’ and ‘papers out’. As it turns out, neither of these are good measures of research quality*”

“So what should we do?”

“*Well Prof. Benchie, I can't sort this out by myself, and I don't think you can sort it out by yourself either. But together, learning from the strengths and weaknesses of our respective backgrounds, we should be able to make things a little better, a little bit more reliable, to make the process of translation a little bit more systematic. And as we revel in the camaraderie and enjoyment of accumulating lots of little bits of improvement, we might end up with quite a lot of improvement*”

“That, Athena, sounds like the starting point for an exciting journey. Do you mind if I join you?”

## ENDNOTE

1 “Looking for the pony” comes from a Christmas tale of two brothers, one of whom was incurable pessimist and the other, an incurable optimist. On Christmas day, the pessimist was given a roomful of shiny toys and the optimist, a roomful of horseshit. The pessimist opened the door to his roomful of toys, sighed, and lamented, “A lot of these are motor driven and their batteries will run down; and I suppose I'll have to show them to my cousins, who'll break some and steal others; and their paint will chip; and they'll wear out. All in all, I wish you hadn't given me this roomful of toys.” The optimist opened the door to his roomful of horseshit and, with a whoop of glee, threw himself into the muck, and began burrowing through it. When his horrified parents extracted his from the excrement and asked him why on earth he was thrashing about in it, he joyfully cried: ”With all this horse shit, there's got to be a pony in here somewhere!”
